# Statins for Patients Undergoing Thoracic Aortic Aneurysm Repair Surgery: What to Do?

**DOI:** 10.1055/s-0041-1736589

**Published:** 2021-12-03

**Authors:** Katherine M. Klein, Ion S. Jovin

**Affiliations:** 1 Department of Surgery/Cardiothoracic Surgery, Virginia Commonwealth University, Richmond, Virginia; 2 Department of Medicine/Cardiology, Virginia Commonwealth University, Richmond, Virginia; 3 Medical Service, McGuire Veterans' Administration Medical Center, Richmond, Virginia

**Keywords:** statins, thoracic aortic aneurysm, aneurysm repair, outcomes

## Abstract

Statins may be associated with improved outcomes in patient with thoracic aortic aneurysms but there is little data on the role of statins in patients who have undergone thoracic aortic aneurysm repair.

Since their introduction in clinical practice in the late 1980s, statins have become widely used in the primary and secondary prevention therapy of patients with cardiovascular risk factors and patients with cardiovascular disease, respectively. 1 2 3 More recently published data suggest that statins also have a role in the treatment of patients with aortic aneurysms where they have been shown in smaller retrospective studies to be associated with a reduced number of events such as dissection, rupture, and death. 4 5 6 7

In this issue of the journal, Kindzelski et al 8 report on the association between statin therapy and outcomes in a cohort of patients who underwent thoracic aortic aneurysm surgery. The study did not find any survival advantages with statin therapy and the authors conclude that statins should not be prescribed in patients with thoracic aortic aneurysms unless there is an evidence-based indication for therapy other than the thoracic aortic aneurysm. The paper is intriguing because of the scarcity of data on this type of patients, but it has some significant issues: the authors only examined the outcomes of patients after surgery and not the course before surgery. Therefore, based on their data, they can only make inferences regarding patients who have undergone thoracic aortic surgery and not all patients with thoracic aortic aneurysm.

As the authors correctly point out in the discussion, this report does not provide data regarding aneurysm growth rate and frequency of rupture or dissection. Moreover, the authors need to state clearly that, even with propensity matching, the study is a retrospective study and as such should be regarded as hypothesis-generating only. While the authors are to be commended for this study which provides data about the utility of statins in the postoperative period, it seems that these data suggest that there is no additional benefit of statins in postoperative patients who have undergone thoracic aortic surgery. Furthermore, a definitive study will be a randomized trial that looks at this specific question and that patients who already are on a statin should not stop therapy based on these retrospective data alone, especially since data on patients with both abdominal and thoracic aortic aneurysms from Taiwan seem to conflict with the findings of the authors regarding mortality. 7 Finally, a perhaps philosophical or semantic question is the question whether a patient with an aneurysm rupture or dissection should be considered as having had a cardiovascular event or not. 9

The authors also do not support their statement that “Nonetheless, there is widespread prophylactic use of statins in patients with thoracic aorta disease in the absence of well-established cardiovascular indications” with any references and we do not really know what proportion of the use of statins is prophylactic for preventing aneurysm-related complications and what proportion is guideline-directed medical therapy for preventions of cardiovascular disease. This is further complicated by the fact that over time the serum cholesterol targets have decreased 10 and thus the time of the aortic surgery may influence the classification of statin therapy, with a patient treated in 2005 having a different low-density lipoprotein cholesterol target than a patient treated in 2011. The inference that patients who did not have cardiovascular indications were receiving statins for their thoracic aneurysm is not confirmed.

The authors acknowledge that the issue of statins in the perioperative period in patients undergoing cardiovascular surgery 11 12 is incompletely understood, especially the relationship between statins and postoperative acute kidney injury 13 14 but they should also acknowledge that the results of this study can only be considered hypothesis-generating. Since this is not a randomized clinical trial, despite the propensity score matching, some hidden biases may still be present and, with the potential for misclassification of prophylactic therapy with statins as outlined above, the conclusions of the authors are open to challenge. Moreover, their findings do not pertain to all patients with thoracic aortic aneurysms: the findings of the paper only pertain to patients who underwent thoracic aneurysm surgery.

With all the limitations mentioned above, the value of the paper by Kindzelski et al ^8^ should not be questioned. However, what is the take home message of the paper? The fact that statins may not be universally beneficial and that patients who have undergone thoracic aortic surgery should be started on a statin only based on an indication for primary or secondary cardiovascular prevention until more data are available is probably a reasonable conclusion. The data in this paper do not justify stopping statin therapy in patients before or after thoracic aortic aneurysm surgery until the prophylactic therapy criteria are better defined and perhaps validated in an external cohort or until a randomized controlled trial addresses this specific question.
